# Tuning the size of quantum dots to enhance charge transfer and photocatalytic CO_2_ reduction

**DOI:** 10.1039/d5ra08597g

**Published:** 2026-04-13

**Authors:** Muhammad Adnan Khalid, Muhammad Mubeen, Muhammad Nasir Hussain, Maria Mukhtar, Amna Iqbal, Sergey A. Kovalenko, Samuel Palato, Baljinder K. Kandola, Azhar Iqbal

**Affiliations:** a Department of Chemistry, Quaid-I-Azam University Islamabad-45320 Pakistan aiqbal@qau.edu.pk; b Institute of Chemistry, Humboldt University of zu Berlin 12489 Berlin Germany; c Institute for Materials Research and Innovation, University of Greater Manchester Bolton BL3 5AB UK

## Abstract

Coupling graphene oxide (GO) with functionalized CdS quantum dots (QDs) can form a promising assembly for the photocatalytic reduction of CO_2_. The functionalizing ligand mercaptoacetic acid (MAA) controls the size of the QDs as well as assists in linking to the surface of GO through the polar groups present on both the QDs and GO. Steady-state photoluminescence (SSPL) and time-resolved photoluminescence (TRPL) analyses reveal photoluminescence (PL) quenching of QDs and suggest the photoexcited charge transfer from QDs to GO. Ultrafast femtosecond transient absorption (TA) spectroscopy corroborates the process of charge transfer in the QDs and GO assembly. Cyclic voltammetry (CV) analysis reveals the difference in the energy levels of QDs and GO, which favors the photoexcited electron transfer from the QDs to GO in the assembly. Electrochemical impedance spectroscopy (EIS) also provides evidence for electron transfer and suppression of electron–hole pair recombination in the assembly. It is found that, among the various QDs-GO assemblies, those with the smallest QDs exhibit the highest charge-transfer efficiency (∼36%) and charge-transfer rate (1.83 × 10^7^ s^−1^). It is further found that the photocatalytic conversion of CO_2_ into formaldehyde strongly depends on the size of the QDs in the QDs-GO assembly. The small QDs assembly exhibits higher photocatalytic performance towards CO_2_ reduction than the large QDs assembly. These findings suggest that QDs-GO assemblies with small QDs facilitate the charge-carrier separation and enable carriers to be available for CO_2_ reduction.

## Abstract 2

Coupling graphene oxide (GO) with functionalized CdS quantum dots (QDs) can form a promising assembly for the photocatalytic reduction of CO_2_.

## Graphical abstract

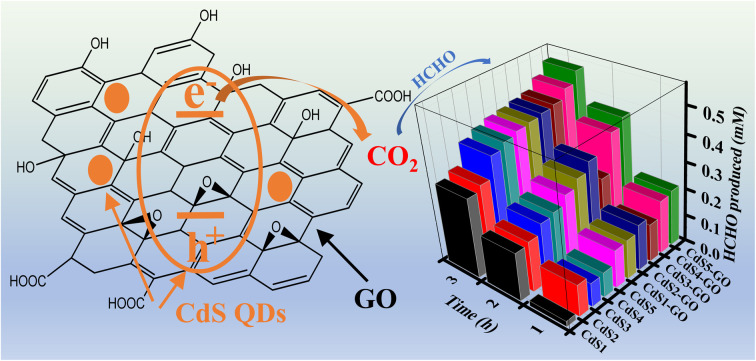

## 1. Introduction

Concerns over climate change have grown over the past few decades due to continuous increase in atmospheric CO_2_ brought about by human activities and industries.^1,2^ One promising strategy to address both the energy issues and the greenhouse effect is photocatalytic CO_2_ reduction.^3–6^ A potential solution to the fossil fuel dilemma is the conversion of CO_2_ into high-value products.^7–10^ Colloidal semiconductor quantum dots (QDs) have great potential for photocatalytic CO_2_ reduction^11–14^ due to their size-dependent tunable characteristics, such as prolonged lifetime emission,^15^ strong luminescence, high fluorescence quantum yield,^16^ and broad absorption and narrow emission spectra. Charge separation, migration, and surface reaction are basic steps in photocatalysis reactions, which depend on the structural and electronic properties of the photocatalyst.^17^ Colloidal QDs are considered potential candidates that can fulfill this series of processes in photocatalysis.

CdS QDs, which are composed of elements cadmium and sulfur from groups II and VI of the periodic table, have a band gap of 2.42 eV (bulk form).^18,19^ As a result of the quantum confinement effect, the CdS QDs exhibit size-dependent electrical, optical, thermal, and PL properties.^20^ Due to their unique optoelectronic characteristics,^21^ CdS QDs have recently gained more attention for applications in a wide range of uses, including solar cells,^22^ LEDs,^23^ photocatalysis,^24^ photodetectors,^25,26^ biolabeling,^27^ fluorescent biological and chemical sensors,^28^ lasers,^29^ optical strain gauges,^29^ and photocatalytic CO_2_ reduction.^30–35^ CdS QDs are considered an attractive photocatalyst for CO_2_ reduction because their conduction band edge position provides electrons with a higher reduction potential under visible-light irradiation.^36–38^ However, when exposed to light and air, the CdS QDs may exhibit unstable luminescence due to photo-oxidation at their surface, resulting in low PL emission intensity *via* trap states that promote non-radiative transitions.^29^ They also exhibit aggregation in solution, which restricts their performance in practical applications due to the fast recombination of photoinduced charge carriers.^37,39,40^ The surface states can be controlled by using surface ligands that passivate the surface of the QDs. For this purpose, many researchers have used thiol group-containing capping ligands such as MAA and 3-mercaptoperpionic acid (MPA).^32,41^ By using “capping” molecules, the emission wavelength may also be adjusted. The optical, chemical, and photophysical properties, such as enhancement of emission intensity and improvement in the photostability of CdS QDs, may be changed due to surface modification. This modification causes the generation of new trap states on the surface of the QDs, resulting in the appearance of new emission bands and enhancing the stability and selectivity of the QDs. The addition of organic ligands to their surface results in both the stabilization of the QDs in various solvents and the desired surface functionality. In addition to the surface functionalization strategy of CdS QDs, the best way to minimize charge recombination is to construct the heterostructure/assembly with two-dimensional (2D) materials. In this scenario, CdS QDs have been decorated on various 2D materials, especially carbon-based materials such as carbon nitrides,^42^ graphene,^43,44^ graphene oxide^45^ and reduced graphene oxide,^46–48^ to construct heterojunctions that separate charge carriers for longer times.^35^

Ultrathin 2D nanosheets frequently exhibit unique physicochemical properties and provide a flexible framework for assembling various nanocomposites with desirable optical, electrical,^49^ and catalytic properties, among other advantages.^50,51^ For example, graphene, an atomic sheet made up of sp^2^-bonded carbon atoms organized in a honeycomb pattern, has been intensively studied for use in energy devices.^52–54^ Due to its high surface area, unique electron reserves and electron-transfer features, superior mechanical properties, high electrical conductivity, as well as excellent electrochemical stability, graphene is a promising candidate for a wide range of applications, such as solar cells, photocatalysis, sensors, light-to-energy conversion, and storage devices.^55,56^ Pure graphene exhibits semi-metal behavior due to its zero-band gap.^57,58^ However, it has poor dispersion in common solvents and aggregates readily. To overcome these challenges, researchers are exploring strategies to functionalize graphene or develop its derivatives, such as GO and rGO. Their size and chemical surface groups need to be carefully governed to render these materials luminous.^59^ GO is a better candidate as an electron acceptor than rGO or even pure graphene, but there are also two drawbacks to using GO: its low absorption coefficient and the fast recombination rate of the charge carrier.^60^ To address these issues, QDs can be deposited on the surface of GO to construct QDs-GO assemblies. Charge-carrier separation takes place at the junction of the assemblies and is essential for effective solar energy conversion. When the assemblies have the appropriate band energy alignment,^61^ charge separation is most effective.^62–64^

In this study, we present the photocatalytic reduction of CO_2_ to formaldehyde mediated by charge transfer in CdS QDs-GO assemblies capped with different concentrations of the MAA ligand. Formaldehyde is a useful industrial intermediate and is used extensively in resin and polymer manufacturing, adhesives and chemical feed. Thus, the photocatalysis of CO_2_ to generate formaldehyde can be viewed as a desirable approach to not only mitigating carbon but also producing value-added chemicals *via* solar energy. MAA not only controls the size but also passivates the surface of CdS QDs and incorporates different surface functionalities that facilitate attachment to GO. The functionalizing ligand MAA coordinates to the surface of CdS QDs through the thiol functional group and changes both the electronic structure and reactivity of the QDs. The charge-transfer process is improved by selecting differently sized CdS QDs functionalized with MAA in the QDs-GO assemblies. The GO-supported QD assemblies enhance photoexcited charge-carrier separation and charge-transfer processes as well as suppress charge recombination. The as-prepared QDs-GO assemblies exhibit high photocatalytic performance for the conversion of CO_2_ into formaldehyde.

## 2. Experimental

### 2.1. Chemicals

The chemicals used for chemical reactions were CdCl_2_·H_2_O (99%), Na_2_S·9H_2_O (99%), NaOH (99.5%), KOH (95%), KMnO_4_ (99%), NaNO_3_ (97%), H_2_O_2_ (30% weight in H_2_O), HCl (98%), CH_3_CO_2_NH_4_ (98%), HCHO (37%) and C_2_H_4_O_2_S (98%). All chemicals were purchased from Sigma-Aldrich and used without any further treatment.

### 2.2. Synthesis of GO, CdS QDs, and QDs-GO assemblies

A modified Hummer's method was used to synthesize GO from graphite powder, as reported in our previous work,^65^ and the CdS QDs were also prepared by following the procedure given in our previous works.^65–67^ Briefly, 10 mL (0.1 mM) of CdCl_2_·5H_2_O and 0.25 M of MAA solutions were prepared in deionized water. The mixture was poured into a three-neck flask and heated to 80 °C, with constant stirring at 300 rpm. The pH of the reaction mixture was adjusted by dropwise addition of freshly prepared 0.1 M aqueous NaOH. After 30 minutes of stirring under an N_2_ atmosphere, 10 mL (0.1 mM) of Na_2_S·9H_2_O was added to the three-neck flask. The reaction mixture was heated and stirred in the same manner for one hour. The resultant CdS QDs were extracted by adding acetone, followed by centrifugation to give the isolated QDs, which was named CdS1. The same procedure was repeated for 0.5 M, 0.75 M, 1 M, and 1.25 M concentrations of MAA, with the same concentration of cadmium precursor, to obtain different-sized QDs named CdS2, CdS3, CdS4 and CdS5, respectively.

To fabricate the CdS QDs-GO assemblies, CdS QDs (1 mg) were dispersed in deionized water (10 mL) and mixed separately with an aqueous suspension of GO (1 mg/10 mL) and stirred for 30 minutes at 60 °C. The resultant mixtures were used for the optical measurements.

### 2.3. Preparation of the photocatalyst for CO_2_

A 0.1 M solution of Na_2_CO_3_ (0.265 g in 25 mL of deionized water) was prepared in a beaker. Afterward, 5 mL of 2-propanol (sacrificial agent) and 25 mL of acetonitrile were added to a 3-neck round-bottom flask sealed with a rubber septum. The as-prepared Na_2_CO_3_ solution was added to the above solution, followed by addition of 0.07 g of the photocatalyst (CdS QDs or CdS QDs-GO). The solution was bubbled with CO_2_ for almost 30 minutes to saturate the solution with CO_2_ completely. The mixture was then illuminated under sunlight for three hours with a solar intensity of ∼200 W m^−2^ and a UV-index of 8.5–8.6 for 3 hours.^68^ A 3 mL sample was taken and added to a glass vial every hour and then treated with freshly prepared Nash's reagent to estimate the amount of formaldehyde produced.

### 2.4. Preparation of Nash's reagent

The amount of formaldehyde produced during CO_2_ photoreduction was determined by treating it with Nash's reagent,^69^ which was prepared by dissolving 0.2 mL of acetylacetone (0.02 M), 0.3 mL of acetic acid (0.05 M), and 1.5 g of ammonium acetate (2 M) in 10 mL of deionized water. The reagent was placed in the dark to avoid any photochemical reaction. The samples (3 mL), which were collected during CO_2_ reduction, were treated with 3 mL of Nash's reagent and heated at 60 °C under constant stirring of 400 rpm for about 10–15 minutes. Consequently, a bright-yellow color developed in the reaction mixture due to the formation of the diacetyldihydrolutidine (DDL) complex. To design a calibration curve for the reference formaldehyde, 10 solutions of known molarities (0.05 mM, 0.10 mM, 0.15 mM, 0.20 mM, 0.25 mM, 0.30 mM, 0.35 mM, 0.40 mM, 0.45 mM, and 0.50 mM) were also prepared and treated with Nash's reagent in the same way. The optical density was measured at *λ*_max_ = 412 nm using a UV-Vis spectrometer. The formaldehyde concentration was then calculated using the constructed calibration curve.

### 2.5. Characterization techniques

Fourier-transform infrared (FTIR) analysis was performed using a Bruker Tensor II spectrophotometer. X-ray diffraction (XRD) (PANalytical X-ray diffractometer) analysis was used to estimate the phase of the QDs and GO. A Shimadzu UV-1601 spectrophotometer was used to measure the absorption spectra of the QDs and their assemblies. A JEM-1200 EX MKII was used to perform energy-dispersive X-ray (EDX) analysis and for transmission electron microscopy (TEM). The photoinduced charge separation and recombination process in QDs and their assemblies were measured by using a PicoQuant FluoTime 300 spectrophotometer, as described in our previous work,^70–74^ and a homebuilt TA spectrometer, as described by Dobryakov *et al.*^75^ The excitation pulse for transient measurements was generated by using an optical parametric amplifier and frequency quadrupling its output (light conversion, TOPAS prime with NIRUVIS). The probe spectrum was generated by supercontinuum generation in a calcium fluoride plate driven at 400 nm. TA spectra were measured in the spectral range 275–690 nm with a 0.4-mm thick flow cell. The magic-angle signal Δ*A*(*λ*, *t*) was obtained from parallel and perpendicular pump-probe polarization measurements as Δ*A* = (2Δ*A*_⊥_ + Δ*A*_∥_)/3. The temporal instrument response was 0.1 ps broad. Multiple 10–20 pump-probe scans were applied to improve the signal-to-noise ratio. Measurements were carried out using 360 nm and 420 nm excitation wavelengths, yielding consistent results. A Gamry Instruments Interface 1010E was used for cyclic voltammetry (CV) analysis. To fabricate the working electrode, approximately 1 mg of each material was deposited onto nickel foam. Ag/AgCl and Pt foil were utilized as the reference and counter electrodes, respectively. The CV experiments are conducted within a working potential range of −0.2 V to +0.6 V, employing a 3 M KOH aqueous electrolyte at a scan rate of 10 mV s^−1^.

## 3. Results and discussion

**Fig. 1:**
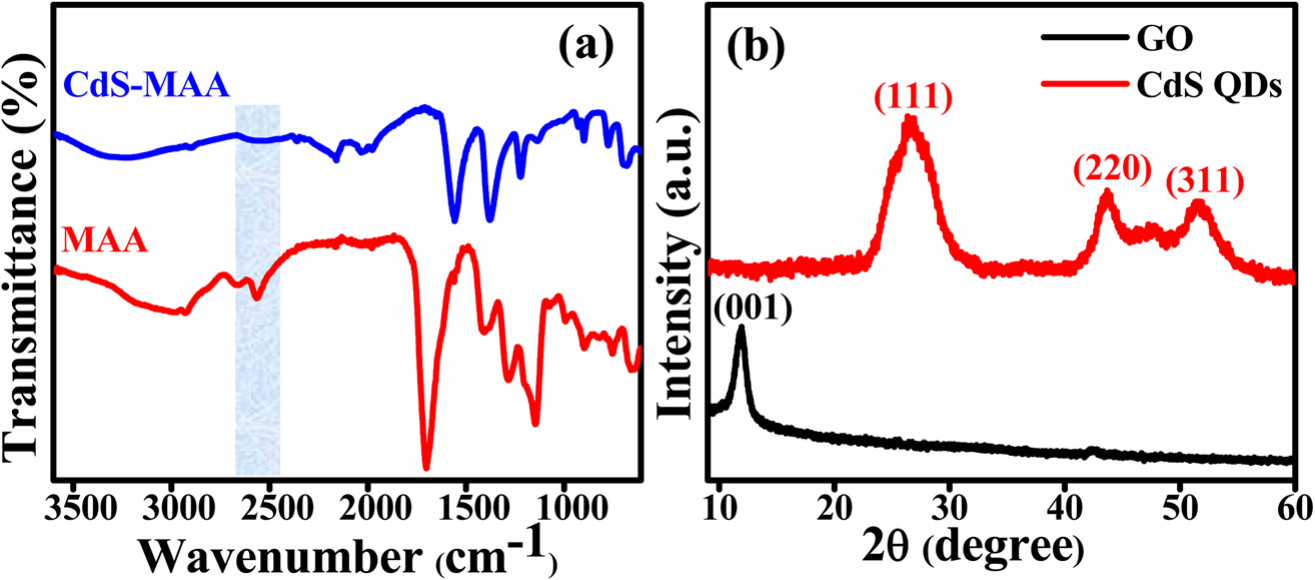
(a) FTIR spectra of MAA and MAA-functionalized CdS5 QDs, and (b) XRD patterns of GO and CdS5 QDs.

FTIR analysis was employed to verify the anchoring of MAA to the surface of the CdS QDs, as depicted in Fig. 1(a). The distinctive signals at 668 cm^−1^, 1658 cm^−1^, and 3350 cm^−1^ are attributed to the presence of the Cd–S, C=O, and O–H bonds, respectively.^18,76–78^ The absence of any characteristic peak in the region between 2500–2650 cm^−1^ and the presence of a broad peak in the region between 3100 and 3400 cm^−1^ indicate that there is no S–H stretching band present, which indicates the attachment of MAA to the surface of QDs *via* the thiol moiety. In the XRD pattern of the CdS QDs (Fig. 1(b)), three distinct peaks appear at 2*θ* values of 27°, 43°, and 52°, corresponding to the (111), (220), and (311) reflection planes, respectively.^79^ The face-centered cubic crystal structure of the synthesized CdS QDs is confirmed by comparison with the JCPDS card no. 00-010-0454.^67^ The peak broadening of the (111) plane indicates that the size of the synthesized QDs is approximately 2.5 nm, estimated from Scherrer's formula.^80^ The diffraction peak at 10.8° corresponds to the (001) plane of GO.^81^

**Fig. 2:**
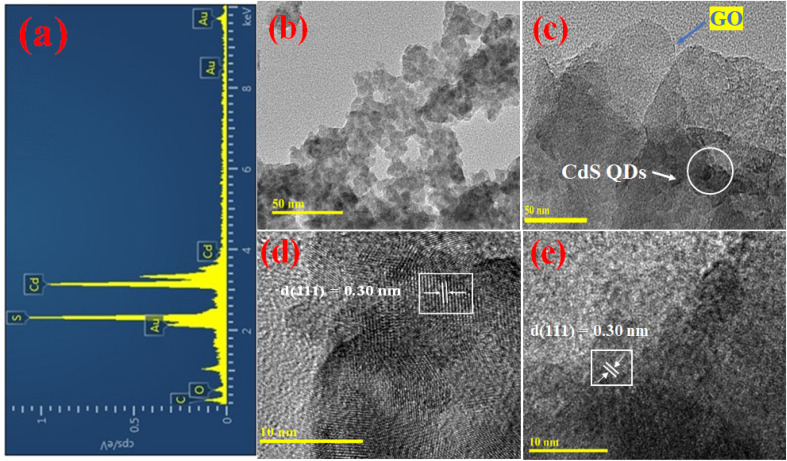
(a) EDX of CdS5 QDs. (b) TEM micrograph of CdS5 QDs. (c) TEM micrograph of the assembly. (d) HRTEM micrograph of CdS5 QDs. (e) HRTEM micrograph of the assembly.

The elemental purity of the synthesized CdS QDs was demonstrated by EDX analysis. The Cd and S peaks are dominant (Fig. 2(a)). The carbon and oxygen peaks are attributed to the existence of the functionalizing ligand MAA used during the synthesis of CdS QDs. As seen in the TEM image (Fig. 2(b)), the QDs are spherical and have sizes of less than 5 nm. The TEM image of the assembly shows the existence of both QDs and GO (Fig. 2(c)). The image clearly shows the porosity and the size homogeneity. An inter-planar lattice fringe spacing of 0.30 nm is shown in a high-resolution TEM micrograph (Fig. 2(c) and (d)), which correlates to the (111) plane of the CdS QDs.

**Fig. 3:**
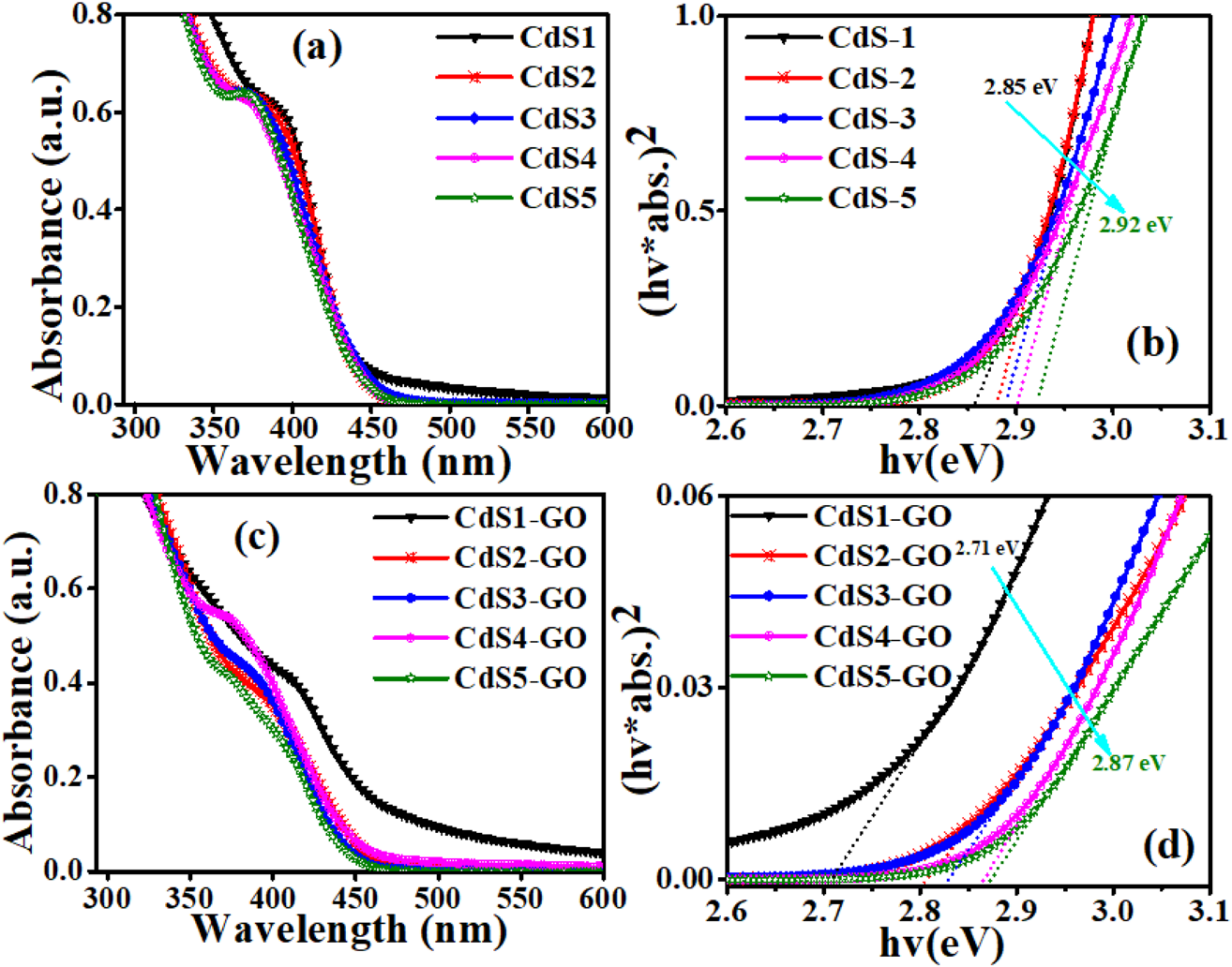
(a) UV-Vis spectra of the CdS QDs. (b) Tauc plots of the CdS QDs. (c) UV-Vis spectra of the CdS-GO assemblies, and (d) Tauc plot of the CdS-GO assemblies.

The UV-Vis analysis depicted in Fig. 3(a) shows the first excitonic peak of CdS QDs at 394 nm, 384 nm, 376 nm, 372 nm, and 368 nm for CdS1, CdS2, CdS3, CdS4, and CdS5 samples, respectively, where suffixes 1–5 denote the sizes in decreasing order. The band gap estimated by Tauc plot corresponds to these absorption edges of 2.85 eV, 2.88 eV, 2.89 eV, 2.90 eV, and 2.92 eV, respectively (Fig. 3(b)). The blue shift in absorption wavelength and high band gap values of the CdS QDs indicate the existence of quantum confinement due to the capping ability of the functionalizing ligand. By increasing the concentration of MAA, the QDs exhibit absorptions at shorter wavelengths, suggesting the size distribution of QDs estimated by Peng's equation,^67,82–84^ as shown in Table 1. An effective mass model approximation (Brus equation)^80^ was also used to determine the average particle size based on the estimated band gap values obtained from the UV-Vis absorption spectra of the CdS QDs (Table 1). The calculated size based on the Brus equation is different from that based on the Peng equation because the two equations have different approximation models and involve different numbers of factors. However, the size calculated by the Peng equation shows good agreement with the XRD analysis results.

**Table 1:** CdS QD size estimated using the Peng and Brus equations

Sample	QD size (nm) Peng equation	QD size (nm) Brus equation
CdS1	3.19	3.56
CdS2	2.89	3.46
CdS3	2.68	3.44
CdS4	2.57	3.40
CdS5	2.47	3.34

**Fig. 4:**
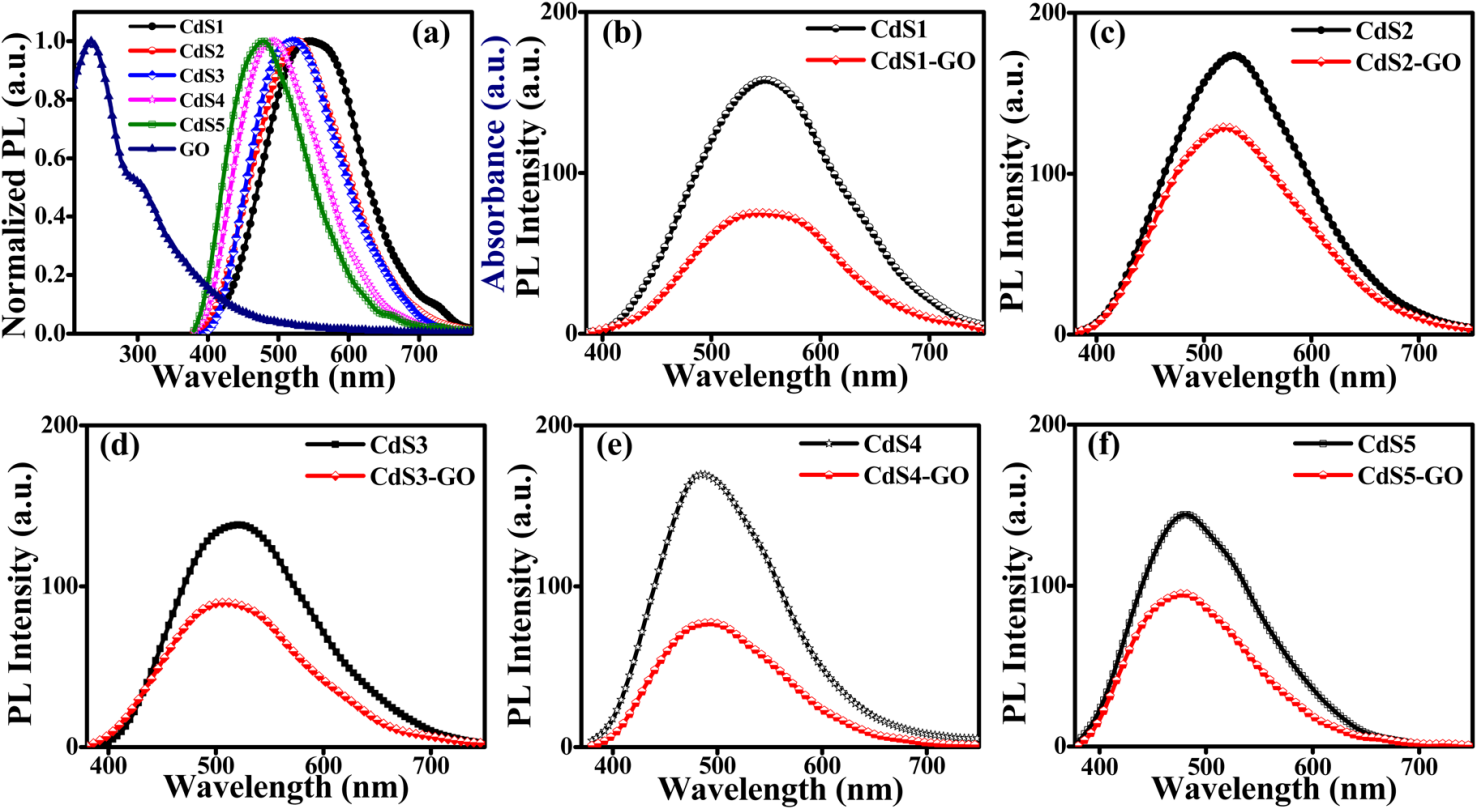
(a) Normalized PL of QDs and absorption spectrum of GO. (b) PL quenching of CdS1 QDs, (c) CdS2 QDs, (d) CdS3 QDs, (e) CdS4 QDs and (f) CdS5 QDs in the absence and presence of GO.

The UV-Vis spectra (Fig. 3(c)) of CdS1-GO, CdS2-GO, CdS3-GO, CdS4-GO, and CdS5-GO assemblies display absorption edges at 413 nm, 396 nm, 388 nm, 375 nm, and 372 nm, respectively. GO shows the first excitonic peak around 235 nm (Fig. 4(a)) along with a weaker shoulder peak at 305 nm. In comparison to the pristine CdS QDs, the absorption spectra of the CdS-GO assemblies are redshifted. The redshift in assemblies is due to the addition of GO, the carbon content of which absorbs the light and alters the surface of the CdS QDs.^82,85^ GO not only has high electron mobility but also suppresses the recovery of electrons and holes.^86^ The addition of graphene changed the electron-transport ability of CdS QDs. The band gaps of the assemblies estimated by Tauc's method are 2.71–2.85 eV (Fig. 3(d)).

The PL spectra were recorded after pulsed excitation at 306 nm of MAA functionalized CdS QDs and their assemblies with GO (Fig. 4). The PL emission spectra shift toward shorter wavelengths (from 555 nm to 480 nm) with increasing concentration of capping ligand (Fig. 4(a)). These results are consistent with the UV-Vis measurements (Fig. 3) and show the successful capping ability of the capping ligands. The assemblies of these QDs with GO show a decline in their PL intensity. This steady decline in the PL intensity indicates a reduction in the radiative recombination of electrons and holes in the assemblies. This suggests the occurrence of charge transfer from the CdS QDs to GO. It means the addition of GO to the CdS QDs enhances the electron transport in the assemblies and helps to reduce the charge recombination process, which leads to a higher photocatalysis rate.^87^

**Fig. 5:**
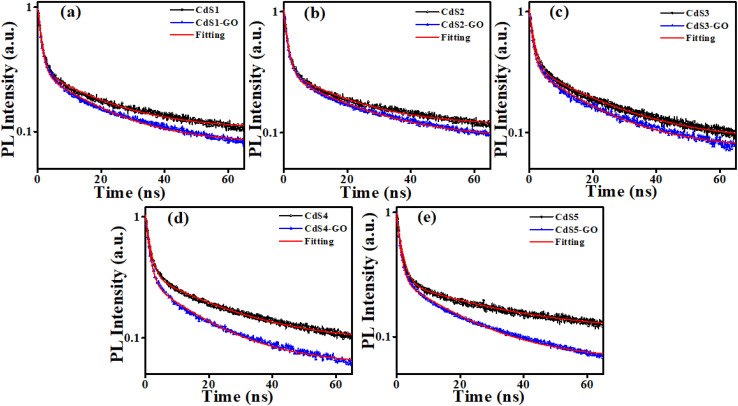
(a) PL decay kinetics of CdS1 QDs, (b) CdS2 QDs, (c) CdS3 QDs, (d) CdS4 QDs, and (e) CdS5 QDs in the absence and presence of GO.

Following excitation at 306 nm, PL decay kinetics measurements were recorded, as shown in Fig. 5. The PL decay kinetics are shown in different color traces, with red indicating the best fit achieved by the bi-exponential decay model. The average measured PL lifetimes determined using eqn (1)^60^ of CdS1, CdS2, CdS3, CdS4, and CdS5 QDs are 19.89 ns, 21.81 ns, 22.05 ns, 23.01 ns, and 30.99 ns, respectively. After the addition of GO, the decline in the PL lifetime of CdS1-GO, CdS2-GO, CdS3-GO, CdS4-GO, and CdS5-GO assemblies is also observed, with the corresponding values of 18.10 ns, 19.31 ns, 18.27 ns, 17.76 ns, and 19.77 ns, respectively. This shortening of the PL lifetime of the assemblies indicates the electron transfer from the QDs to GO, as evidenced by cyclovoltammetry measurements discussed in the next section. The CdS5 QDs and their assemblies, which are functionalized with a higher concentration of capping ligand MAA and have a small size, show a longer lifetime as compared to the other QDs and their assemblies. This can be attributed to the small QDs having relatively large band-gap values, enhanced surface area, and improved charge-carrier separation. The higher band gap of the small QDs means the conduction band is higher than the lowest unoccupied molecular orbital (LUMO) of GO, and electron transfer is faster.^88^ The PL kinetics results further support the strong influence of surface ligands on charge-carrier dynamics. The longer PL lifetime observed for smaller QDs may arise from ligand-induced hole trapping, consistent with reports that surface-thiol and carboxylates can introduce mid-gap states. Such traps enforce charge separation, thereby modulating the efficiency of CO_2_ photoreduction, as discussed below. The correlation between spectroscopic signatures and catalytic performance indicates that the electronic structure is not only size-dependent but also strongly influenced by the surface functionalization.^89,90^ The calculated electron-transfer rate (*k*_CT_) and electron-transfer efficiency (*E*_CT_), using eqn (2) and (3),^70,71,73^ are given in Table 2.1
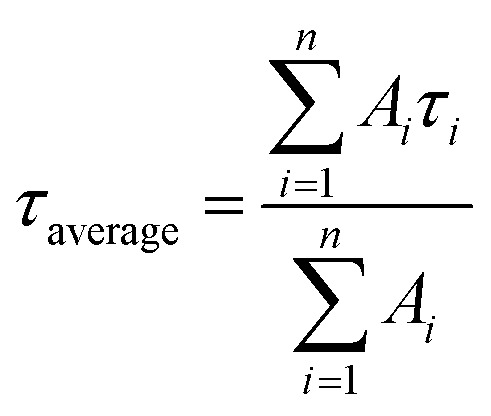
2
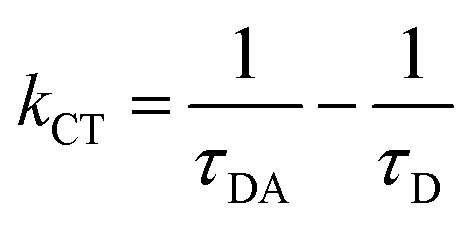
3
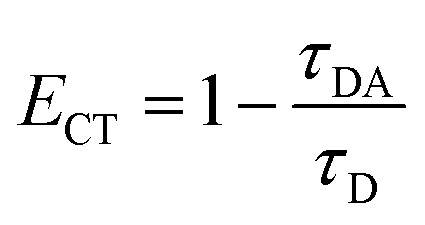
where the *τ*_D_ and *τ*_DA_ are the PL lifetimes of the CdS QDs and their assemblies with GO, respectively. The CdS5 QDs and their assemblies show the maximum values of electron-transfer rate (1.83 × 10^7^ s^−1^) and electron-transfer efficiency ∼36% (see Table 2). This means that the charge-transfer efficiency is dependent upon the size of the donor QDs.

**Table 2:** Average PL lifetime, *k*_CT_, and *E*_CT_ of CdS QDs and their assemblies

Samples	*τ*_average_ (ns)	*k*_CT_ (s^−1^)	*E*_CT_ (%)
CdS1 QDs	19.89	—	—
CdS1-GO	18.10	4.97 × 10^6^	9.00
CdS2 QDs	21.81	—	—
CdS2-GO	19.31	5.93 × 10^6^	11.46
CdS3 QDs	22.05	—	—
CdS3-GO	18.27	9.38 × 10^6^	17.14
CdS4 QDs	23.07	—	—
CdS4-GO	17.76	1.29 × 10^7^	23.01
CdS5 QDs	30.99	—	—
CdS5-GO	19.77	1.83 × 10^7^	36.20

**Fig. 6:**
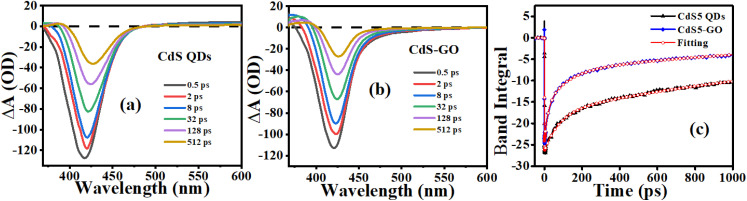
fs-TA spectra of CdS5 QDs (a) and the CdS5-GO assembly (b) with selected time delays, and (c) TA decay kinetics of CdS QD in the absence and presence of GO at *λ*_pump_ = 360 nm.

We further studied the electron-transfer dynamics in CdS QDs and CdS-GO assemblies using femtosecond transient absorption (fs-TA) spectroscopy. The TA spectra of the CdS5 QDs and CdS5-GO assembly for selected time delays are shown in Fig. 6(a) and (b), respectively. The negative peak around 420 nm is due to the ground-state bleach (GSB) signals of the CdS QDs. The GSB signal is due to the presence of a core exciton in the QD. Similar GSB signals to CdS QDs are seen in the CdS-GO assembly spectrum. The positive signal for CdS around 480–660 nm is due to excited-state absorption (ESA) signals. A similar ESA continuum at longer wavelengths than the exciton peak has been assigned to trapped charges at the surface in CdS QDs. The surface charges polarize the QDs and relax the selection rules, thereby allowing the transition from the core exciton to the continuum of states.^91^ This signal is absent in CdS-GO, suggesting that the material does not contain trapped charge carriers.^92,93^

This observation shows GO is coupled to these surface states and acts either statically, by passivating surface traps, or dynamically, by accepting carriers. In either case, the TA spectra confirm an interaction between CdS and GO. To quantify the kinetics, we computed the band integral of the TA spectrum over the GSB peak (BOUNDS) and fitted the resulting transients with a bi-exponential model. The band integral is defined as:^94^4
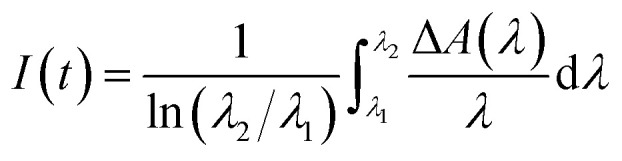


The band integral over the GSB of the exciton peak quantifies the dynamics of the core exciton population. The band integrals and fit results are shown in Fig. 6(c). The lifetimes of CdS5 QDs and CdS5-GO assembly are acquired by the best fit achieved by the bi-exponential decay model. In the case of CdS QDs, the *τ*_1_ (60.63 ps) and *τ*_2_ (680.98 ps) describe the recombination of electrons due to trap states and band-to-band transitions, respectively. For the CdS-GO assembly, the *τ*_1_ (34.23 ps) and *τ*_2_ (338.59 ps) were shorter compared to those of the CdS QDs.^95,96^ The shortening of the lifetime of the CdS QDs in the assembly indicates the ultrafast electron transfer from the QDs to GO. The ground-state bleach signal does not disappear entirely, indicating the charge transfer is only available to part of the sample. The TA measurements confirm that CdS-GO has an effect on the exciton dynamics by accepting carriers. In the assembly, GO acts as an acceptor for carriers and suppresses the charge-recombination process. For CdS5-GO, fs-TA yields *τ*_1_ = 34.23 ps, which is associated with an electron transfer rate of approximately ∼2.9 × 10^10^ s^−1^. This is an ultrafast factor attributed to the interfacial electron injection of photoexcited CdS to GO. In Marcus theory, this high rate means that there is good electronic coupling and a positive charge-transfer driving force. By contrast, TRPL gives a value of *k*_CT_ = 1.83 × 10^7^ s^−1^, which is the ensemble-average depopulation of emissive states on the nanosecond scale. TRPL does not measure the inherent injection event but the competition of radiative recombination, nonradiative decay, and charge transfer. The apparent rate difference is therefore physically justified; fs-TA measures the microscopic rate of electron injection, whereas TRPL measures its macroscopic effect of recombination. The combination of the two methods validates the effective interfacial charge separation of the CdS-GO hybrid system.

In order to assess the exact position of the band edges of the QDs and GO, CV techniques were utilized. CV analysis was carried out to evaluate the conduction band (CB) and valence band (VB) energy level edges of the CdS QDs,^97^ along with the lowest unoccupied molecular orbital (LUMO) and highest occupied molecular orbital (HOMO) of GO.

Eqn (5) and (6) were utilized to calculate the CB and VB energy levels, as well as the LUMO and HOMO levels of the CdS QDs and GO:

5 *E* (LUMO) = −*e*[*E*^onset^_red_ + 4.44]

6 *E* (HOMO) = *E* (LUMO) − *E*_0–0_

**Fig. 7:**
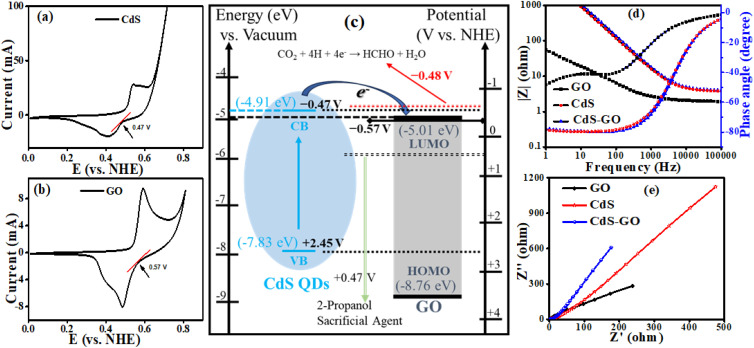
CV profile of (a) CdS QDs and (b) GO, recorded at scan rates of 10 mV s^−1^ in 3 M KOH. (c) Band energies of CdS QDs and GO. (d) Bode plots and (e) Nyquist plots.

The term *E*_0–0_ corresponds to the lowest-energy transition, specifically the 0–0 energy, which is deduced from absorption and emission spectra. Two prominent peaks in the UV-Vis absorption spectra of GO are ascribed to transitions. One is the π → π* transition, manifesting as a peak at 235 nm, and the other is the n–π* transition, peaking at 305 nm. These transitions correspond to the electronic shifts within the C=O and the C=C groups present in GO. The onset reduction potentials of the QDs and GO are determined using the CV (V *vs.* NHE scale) data presented in Fig. 7(a) and (b), resulting in values of 0.47 V and 0.57 V, respectively. The *E*_0–0_ value of QDs obtained from the Tauc plot is 2.92 eV. For QDs, the equation can be modified as:*E* (CB) = −*e*[*E*^onset^_red_ + 4.44]*E* (VB) = *E* (CB) − *E*_0–0_*E* (CB) = −*e*[0.47 + 4.44] eV = −4.91 eV*E* (VB) = [−4.91 − 2.92] eV = −7.83 eV

The obtained values of *E*_0–0_ corresponding to n–π* and π–π* transitions for GO are 3.75 eV and 5.25 eV, respectively.*E*_π*_ (LUMO) = −*e*[0.57 + 4.44] eV = −5.01 eV*E*_nC=O_ (HOMO−1) = [−5.01 − 3.75] eV = −8.76 eV*E*_π_ (HOMO−2) = [−5.01 − 5.25] eV = −10.26 eV

The VB and CB levels of the QDs are positioned at −7.83 eV and −4.91 eV, respectively. In the case of GO, the HOMOs are located at −8.76 eV (associated with nC=O) and −10.32 eV (related to π orbitals), while the LUMO resides at −5.01 eV (π* orbital), as indicated in Fig. 7(c). Upon evaluating the band edge values of both the QDs and GO, it becomes evident that in the assemblies, an electron transfer process is viable from photoexcited CdS QDs to GO. This electron transfer quenches the PL of the QDs in the assemblies. Fig. 7(d) depicts the Bode plots of the GO and CdS QD electrodes. The phase angles observed in these plots suggest the charge-transfer characteristics of the materials. Nyquist plots illustrate the EIS spectra of the CdS QDs, GO, and CdS-GO assembly, as shown in Fig. 7(e). These plots reveal distinctive features: a depressed arc noticeable at high-frequency regions, and a pronounced steep line at lower frequencies. These characteristics are attributed to charge-transfer processes in the materials at the electrode interface. The point of intersection with the real axis of the plot signifies the bulk resistance, with a downward shift indicating a reduction in the electrode's bulk resistance. In comparison, the bulk resistance of GO is notably higher than that of the corresponding CdS QDs. Within the low-frequency range, a steeper line with the imaginary axis suggested a faster rate of charge transfer. Notably, the QDs exhibit higher slope values than the GO electrodes, which also indicates a rapid transfer of electrons from CdS QDs to GO. The smaller arc of the CdS-GO assembly shows a lower charge-transfer resistance of 26.18 ohms. The significant reduction in charge-transfer resistance by decorating the CdS QDs (29.58 ohms) on the surface of GO (34.20 ohms) indicates the good transport ability of the assembly. The assembly's smaller EIS curve radius than those of pristine CdS and GO results in fewer electron and hole recombination events.^55,98,99^

**Fig. 8:**
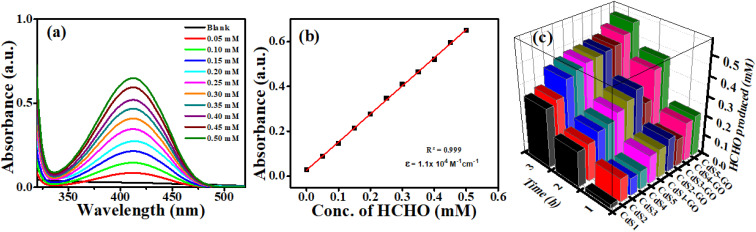
(a) Absorption spectra for known concentrations of formaldehyde, (b) calibration curve for formaldehyde and (c) a comparative study of the photoreduction efficiency of CdS QDs and their assemblies.

The CdS QDs and their assemblies were used as photocatalysts for CO_2_ reduction to formaldehyde. The CdS QDs and GO energy bands are aligned (Fig. 7(c)) on a single vacuum and Normal Hydrogen Electrode (NHE) scale, along with the redox potential of CO_2_/HCHO and 2-propanol (as a sacrificial agent). CdS and GO have onset reduction potentials of −0.47 V and −0.57 V *vs.* NHE, which are equal to −4.87 eV and −4.97 eV *vs.* vacuum, respectively. Since the LUMO of GO is lower than the CB of CdS QDs, it is possible to allow photogenerated electrons to relocate between CdS and GO, thus facilitating charge separation and electron accumulation. Even though the potential of the CO_2_ to HCHO (−0.48 V *vs.* NHE) reduction is near the CdS CB edge, several factors support the reaction. Firstly, these potentials are standard thermodynamic values at equilibrium electrochemical conditions, and photocatalytic reactions take place under non-equilibrium conditions in which photogenerated electrons have additional kinetic energy. Secondly, GO enhances the removal of electrons and their transfer, preventing the recombination process and providing a driving force for CO_2_ reduction. Thirdly, CO_2_ reduction proceeds through a multi-step proton-coupled electron transfer mechanism, in which sequential electron transfer stabilizes high-energy intermediates, thereby lowering the effective activation barrier relative to a direct electron transfer pathway. Moreover, CdS VB (+2.45 V *vs.* NHE) has an overpotential that is high enough to oxidize 2-propanol (+0.47 V *vs.* NHE), and thus, the holes are easily scavenged, so that recombination is effectively inhibited. The formaldehyde is detected by using Nash's reagent. Nash's reagent test is a simple, rapid, economical and convenient method for confirming the presence of formaldehyde formation. No formaldehyde was formed when the experiment was performed in the absence of light or a catalyst (Fig. 8(a)). Product quantification was carried out using the Nash reagent assay, which selectively detects formaldehyde through the formation of a yellow chromophore with maximum absorbance at 412 nm. Owing to the specificity of this method, formaldehyde was identified and quantified as the major reduction product, whereas other possible CO_2_ reduction products were below the detection limit under the present conditions.^100,101^ A calibration curve was constructed by taking known concentrations of formaldehyde to determine the absorptivity coefficient (*ε*) of DDL, and the amount of produced formaldehyde. “*ε*” was found to be 1.1 × 10^4^ M^−1^ cm^−1^ as shown in the absorbance *vs.* wavelength graph of DDL (formed as a result of reaction between known concentrations of formaldehyde and Nash reagent). Similarly, the absorbance *vs.* concentration of the formaldehyde (known) graph was plotted to calculate the amount of formaldehyde (unknown) produced, as shown in Fig. 8(b) and (c). The DDL produced from each sample (withdrawn after regular intervals of 1 hour) was compared at the corresponding time.^102,103^ It is observed that, with increasing irradiation time, the peak of the DDL complex also increases due to the effective production of formaldehyde. By comparing the data in Fig. 8(c), it is seen that the maximum increase in DDL complex peak within 3 hours is shown by the CdS5-GO assembly.

Several notable findings emerged from photoreduction experiments of CO_2_ utilizing CdS QDs. First, we observe a clear trend: as the size of the CdS QDs decreases, the photocatalytic efficiency of the CO_2_ reduction increases due to enhanced surface area and improved charge-carrier separation. The link between QD size and band gap can explain this process because smaller QDs have larger band gaps, and hence, they are more efficient catalysts for converting CO_2_ into formaldehyde. The formation of DDL from formaldehyde produced by each sample is compared at the corresponding time, as shown in the series in Fig. 8(c). In addition, we investigated the effect of the size of the QDs in the assemblies on the photocatalytic reduction of CO_2_.

There are two factors that can be identified as the primary causes of the improved activity of our materials (as summarized in Table 3). The size of the CdS QDs is small enough to increase the surface-to-volume ratio and reduce the diffusion pathways that the charge-carriers follow, thus increasing light absorption and further separating the electron–hole pairs that arise on photogeneration. Moreover, the incorporation of GO creates a conductive interface that facilitates quick electron transfer between CdS and GO, in effect, inhibiting charge recombination. This interfacial charge transfer enhances the accessibility of electrons that can reduce CO_2_, resulting in high photocatalytic activity. Finally, our findings highlight the importance of the impact of the size of the QDs in the QDs-GO assemblies for optimizing CO_2_ reduction efficiency. Efficiency is calculated in terms of activity^104^ by using eqn (7), and is given in Table 3.7
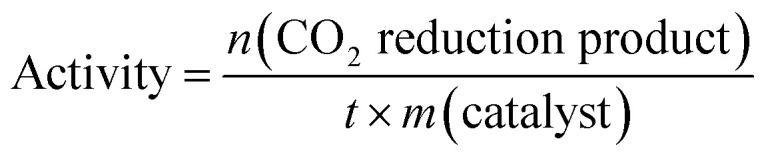
where *n* (CO_2_ reduction product) is the number of moles of formaldehyde produced, *m* is the mass of the catalyst in grams, and *t* is the time in hours.

**Table 3:** Activity of CdS QDs and their assemblies with GO

Sample	Activity (CdS) (µmol g^−1^ h^−1^)	Activity (CdS-GO) (µmol g^−1^ h^−1^)
CdS1	1222	1844
CdS2	1300	1866
CdS3	1588	1927
CdS4	1738	2067
CdS5	1816	2224

Some of the recent reported photocatalysts for CO_2_ reduction make use of expensive, precious-metal-based complexes, others use the metal-doped QDs, QD composites and bare QDs, which involve multiple synthesis steps before the desired product is achieved. Formaldehyde (HCHO), formic acid (HCOOH), methanol (CH_3_OH), carbon monoxide (CO) and methane (CH_4_) are photoproducts in the works, and the photocatalytic efficiency was examined. In comparison to those photocatalysts, the photocatalysts composed of MAA-functionalized CdS QDs and their assemblies with GO, discussed in the present work, were obtained through a simple colloidal synthesis strategy based on the use of usual inexpensive precursors, and they exhibited much better photocatalytic CO_2_ reduction efficiency. A comparison is provided in Table 4. The activities of assemblies with small CdS QDs are highest, which shows that QD size as well as GO addition can have a significant impact on the photocatalytic properties of CdS QDs. It is evident from Table 3 that QDs in the form of assemblies are more efficient photocatalysts compared to bare QDs, and this photocatalytic behavior of QDs is a function of their sizes; *i.e.*, small QDs are more efficient photocatalysts, as evident from their turnover number.

**Table 4:** Comparison of the activity of CdS QDs and their assemblies in the photocatalytic CO_2_ reduction with reported photocatalysts

Catalyst	Product yield (µmol g^−1^ h^−1^)				HCOOH	Reference
	CO	HCHO	CH_4_	CH_3_OH		
3DOMCdSQD/NC	5210					30
ZnCdSe-CdS			240			105
Mg^2+^-doped CdS			45.8			106
FeOOH/CdS	12.55		5.88			107
g-C_3_N_4_/Cu/TiO_2_		5069		2574		108
Co_3_O_4_-TiO_2_/CdS-CuO_*x*_ heterojunction	124					109
0.4CDs/CdS QDs (bio) composites					439.51	110
CdS-InP composites	216					111
MAA capped CdS QDs		2124				112
MAA capped CdS QDs		1816				This work
CdS-GO composite		2224				This work

## 4. Conclusion

The functionalizing ligand MAA controlled the size of the QDs as well as facilitated their attachment to the surface of GO through the interaction of polar groups present on the surface of both QDs and GO. The SSPL and TRPL analyses determine the PL quenching and electron transfer from the QDs to GO in the CdS-GO assemblies. Furthermore, the fs-TA measurements also revealed the ultrafast electron transfer from CdS QDs to GO in the assembly. CV analysis suggested the alignment of CdS QDs and GO energy levels, which favors photoexcited electron transfer from the CdS QDs to GO. The EIS also supports the faster charge transfer and suppression of electron–hole pair recombination in the assemblies. The QDs-GO assemblies with smaller QDs showed higher charge-transfer efficiency compared to other assemblies. These assemblies also exhibited the highest photocatalytic performance for the conversion of CO_2_ into formaldehyde. Our study should inspire future investigations into photoexcited charge transfer and photocatalytic CO_2_ reduction in functionalized QD donors and GO acceptor assemblies.

## Conflicts of interest

There are no conflicts to declare.

## Supplementary Material

RA-016-D5RA08597G-s001

## Data Availability

All data analyzed during this study are included. Supplementary information (SI): TA data following excitation at 360 nm and 420 nm. See DOI: https://doi.org/10.1039/d5ra08597g.

## References

[cit1] Huang J., Li Q., Song Z. (2022). Sci. Total Environ..

[cit2] Zhai Y., Zhang B., Shi R., Zhang S., Liu Y., Wang B., Zhang K., Waterhouse G. I., Zhang T., Lu S. (2022). Adv. Energy Mater..

[cit3] White J. L., Baruch M. F., Pander III J. E., Hu Y., Fortmeyer I. C., Park J. E., Zhang T., Liao K., Gu J., Yan Y. (2015). Chem. Rev..

[cit4] Wang J., Shi Y., Wang Y., Li Z. (2022). ACS Energy Lett..

[cit5] Wang Y., Wang J., Zhang M., Zheng S., Wu J., Zheng T., Jiang G., Li Z. (2023). Nano Micro Small.

[cit6] Li N., Chen X., Wang J., Liang X., Ma L., Jing X., Chen D.-L., Li Z. (2022). ACS Nano.

[cit7] Wu H. L., Li X. B., Tung C. H., Wu L. Z. (2019). Adv. Mater..

[cit8] Izumi Y. (2013). Coord. Chem. Rev..

[cit9] Dhakshinamoorthy A., Navalon S., Corma A., Garcia H. (2012). Energy Environ. Sci..

[cit10] Li C., Xu X., Jia A. (2025). Surf. Interfaces.

[cit11] Habisreutinger S. N., Schmidt-Mende L., Stolarczyk J. K. (2013). Angew. Chem., Int. Ed..

[cit12] Cai J., Li X., Su B., Guo B., Lin X., Xing W., Lu X. F., Wang S. (2025). J. Mater. Sci. Technol..

[cit13] Yuan Z., Liu J., Xiang Y., Jian X., Zhang H., Liu M., Cao R., Hu Y., Gao X. (2025). J. Colloid Interface Sci..

[cit14] Yang H., Zhang Z., Guo Y., Yuan S., Liu X., Luo D., Ye S. (2025). Adv. Funct. Mater..

[cit15] Chang J., Xia H., Wu S., Zhang S. (2014). J. Mater. Chem. C.

[cit16] Kandi D., Mansingh S., Behera A., Parida K. (2021). J. Lumin..

[cit17] Rothfuss A. R., Ayala J. R., Handy J. V., McGranahan C. R., García-Pedraza K. E., Banerjee S., Watson D. F. (2023). ACS Appl. Mater. Interfaces.

[cit18] Wang S., Yu J., Zhao P., Guo S., Han S. (2021). ACS Omega.

[cit19] Eltaweil A. S., Talaat E., Abd El-Monaem E. M., El-Subruiti G. M. (2025). Solid State Chem..

[cit20] Abdallah S., Al-Hosiny N., Badawi A. (2012). J. Nanomater..

[cit21] Bansal A. K., Antolini F., Zhang S., Stroea L., Ortolani L., Lanzi M., Serra E., Allard S., Scherf U., Samuel I. D. W. (2016). J. Phys. Chem. C.

[cit22] Kokal R. K., Bredar A. R., Farnum B. H., Deepa M. (2019). ACS Appl. Nano Mater..

[cit23] Molaei M., Marandi M., Saievar-Iranizad E., Taghavinia N., Liu B., Sun H., Sun X. (2012). J. Lumin..

[cit24] Jin N., Sun Y., Shi W., Wang P., Nagaoka Y., Cai T., Wu R., Dube L., Nyiera H. N., Liu Y. (2023). J. Am. Chem. Soc..

[cit25] Zhang M. K., Liu W. D., Gong Y. P., Liu Q., Chen Z. G. (2022). Adv. Opt. Mater..

[cit26] Dutta A., Medda A., Ghosh S., Sain S., Patra A. (2022). ACS Appl. Nano Mater..

[cit27] Sobhanan J., Rival J. V., Anas A., Shibu E. S., Takano Y., Biju V. (2023). Adv. Drug Deliv. Rev..

[cit28] Masteri-Farahani M., Khademabbasi K. (2018). J. Lumin..

[cit29] Kaur G., Tripathi S. (2014). Mater. Chem. Phys..

[cit30] Wang F., Hou T., Zhao X., Yao W., Fang R., Shen K., Li Y. (2021). Adv. Mater..

[cit31] Wang H.-Y., Hu R., Lei Y.-J., Jia Z.-Y., Hu G.-L., Li C.-B., Gu Q. (2020). Catal. Sci. Technol..

[cit32] Sagdeev D., Shamilov R., Galyametdinov Y. G. (2021). J. Appl. Spectrosc..

[cit33] Liang X., Wang X., Zhang X., Lin S., Ji M., Liu Q., Wang M. (2024). ACS Catal..

[cit34] Gawal P. M., Golder A. K. (2025). ACS Appl. Energy Mater..

[cit35] Gawal P. M., Ishrat J., Bhattacharyya K., Golder A. K. (2025). Langmuir.

[cit36] Cho K. M., Kim K. H., Park K., Kim C., Kim S., Al-Saggaf A., Gereige I., Jung H.-T. (2017). ACS Catal..

[cit37] Zhu Z., Han Y., Chen C., Ding Z., Long J., Hou Y. (2018). ChemCatChem.

[cit38] Yang K., Yang Z., Zhang C., Gu Y., Wei J., Li Z., Ma C., Yang X., Song K., Li Y. (2021). J. Chem. Eng..

[cit39] Prakash J., Kumar P., Saxena N., Pu Z., Chen Z., Tyagi A., Zhang G., Sun S. (2023). J. Mater. Chem. A.

[cit40] Singh A., Sinha A. S. S. (2018). Appl. Surf. Sci..

[cit41] Swathy S., Anand S. K., Mathew M. R., Kumar K. G. (2021). J. Photochem. Photobiol., A.

[cit42] Guo Y., Wu J., Zhang R., Ocran G. A., An Y., Li K., An S., Guo X. (2024). ACS Sustain. Chem. Eng..

[cit43] Li Q., Li X., Wageh S., Al-Ghamdi A. A., Yu J. (2015). Adv. Energy Mater..

[cit44] Xiang Q., Yu J. (2013). J. Phys. Chem. Lett..

[cit45] Luan X., Dai H., Li Q., Xu F., Mai Y. (2022). ACS Appl. Energy Mater..

[cit46] Singh S., Joe A., Ghotekar S., Kumar G., Lokhande P. E., Kumar D., Hossain K., Pant G. (2024). Desalination Water Treat..

[cit47] Wang H., Wan Y., Li B., Ye J., Gan J., Liu J., Liu X., Song X., Zhou W., Li X. (2024). J. Mater. Sci. Technol..

[cit48] Parihar A., Sharma P., Choudhary N. K., Khan R., Gupta A., Sen R. K., Prasad H. C., Ashiq M. (2023). ACS Appl. Bio Mater..

[cit49] Venugopal G., Krishnamoorthy K., Mohan R., Kim S.-J. (2012). Mater. Chem. Phys..

[cit50] Guan Z., Li X., Wu Y., Chen Z., Huang X., Wang D., Yang Q., Liu J., Tian S., Chen X. (2021). J. Chem. Eng..

[cit51] Lim D.-H., Wilcox J. (2012). J. Phys. Chem. C.

[cit52] Tian Y., Yu Z., Cao L., Zhang X. L., Sun C., Wang D.-W. (2021). J. Energy Chem..

[cit53] Liu C., Yu Z., Neff D., Zhamu A., Jang B. Z. (2010). Nano Lett..

[cit54] Yoo E., Kim J., Hosono E., Zhou H.-s., Kudo T., Honma I. (2008). Nano Lett..

[cit55] Xiao F.-X., Miao J., Liu B. (2014). J. Am. Chem. Soc..

[cit56] Xia Y., Cheng B., Fan J., Yu J., Liu G. (2019). Small.

[cit57] Wang B., Song C. (2022). J. Phys.: Conf. Ser..

[cit58] Nandee R., Chowdhury M. A., Shahid A., Hossain N., Rana M. (2022). Results Eng..

[cit59] Zhu S., Song Y., Zhao X., Shao J., Zhang J., Yang B. (2015). Nano Res..

[cit60] Zheng F., Xu W.-L., Jin H.-D., Hao X.-T., Ghiggino K. P. (2015). RSC Adv..

[cit61] Dehghani Z., Jangi S. R. H. (2025). Fuel.

[cit62] Selinsky R. S., Ding Q., Faber M. S., Wright J. C., Jin S. (2013). Chem. Soc. Rev..

[cit63] Li Q., Yang S., Huang Y., Liang Y., Hu C., Wang M., Liu Z., Tai Y., Liu J., Li Y. (2025). J. Mater. Sci. Technol..

[cit64] Chen J., Huang Y., Wan L., Du C., Zhang Y., Xie M. (2025). Mater. Today Catal..

[cit65] Khalid M. A., Mubeen M., Mukhtar M., Siddique Z., Sumreen P., Aydın F., Asil D., Iqbal A. (2023). J. Fluoresc..

[cit66] Javed H., Fatima K., Akhter Z., Nadeem M. A., Siddiq M., Iqbal A. (2016). Proc. R. Soc. A.

[cit67] Mubeen M., Khalid M. A., Mukhtar M., Sumreen P., Gul T., Ul Ain N., Shahrum S., Tabassum M., Ul-Hamid A., Iqbal A. (2022). Photochem. Photobiol..

[cit68] Adnan S., Hayat Khan A., Haider S., Mahmood R. (2012). J. Renewable Sustainable Energy.

[cit69] Hayun H., Harmita K., Pramudita T. B. (2017). Orient. J. Chem..

[cit70] Mubeen M., Khalid M. A., Gul T., Mukhtar M., Ul-Hamid A., Iqbal A. (2022). ACS Omega.

[cit71] Mukhtar M., Bibi S., Erten Ela S., Yavuz C., Mubeen M., Sumreen P., Khalid M. A., Ul-Hamid A., Iqbal A. (2022). J. Phys. Chem. C.

[cit72] Khalid M. A., Mubeen M., Mukhtar M., Sumreen P., Naz B., Aydın F., Asil D., Iqbal A. (2024). Photochem. Photobiol..

[cit73] Sumreen P., Mukhtar M., Khalid M. A., Mubeen M., Kiran L., Iqbal A., Iqbal A. (2024). New J. Chem..

[cit74] Mukhtar M., Mubeen M., Khalid M. A., Sumreen P., Ul-Hamid A., Ela S. E., Iqbal A. J. (2024). J. Mater. Res..

[cit75] Dobryakov A., Kovalenko S. A., Weigel A., Pérez-Lustres J. L., Lange J., Müller A., Ernsting N. (2010). Rev. Sci. Instrum..

[cit76] Mohamed N. B. H., Brahim N. B., Mrad R., Haouari M., Chaâbane R. B., Negrerie M. (2018). Anal. Chim. Acta.

[cit77] Kouhestany R. H., Azizi S. N., Shakeri P., Rahmani S. (2016). Int. Curr. Pharm. J..

[cit78] Farahmandzadeh F., Salehi S., Molaei M., Fallah H., Nejadshafiee V. (2023). J. Fluoresc..

[cit79] Choi C., Zhao F., Hart J. L., Gao Y., Menges F., Rooney C. L., Harmon N. J., Shang B., Xu Z., Suo S. (2023). Angew. Chem., Int. Ed..

[cit80] Mubeen M., ul Ain N., Khalid M. A., Mukhtar M., Naz B., Siddique Z., Ul-Hamid A., Iqbal A. (2023). RSC Adv..

[cit81] Kaushik J., Sharma C., Lamba N. K., Sharma P., Das G. S., Tripathi K. M., Joshi R. K., Sonkar S. K. (2023). Langmuir.

[cit82] Gao P., Liu J., Lee S., Zhang T., Sun D. D. (2012). J. Mater. Chem..

[cit83] Vikraman A. E., Jose A. R., Jacob M., Kumar K. G. (2015). Anal. Methods.

[cit84] Yu W. W., Qu L., Guo W., Peng X. (2003). Chem. Mater..

[cit85] Jia L., Wang D.-H., Huang Y.-X., Xu A.-W., Yu H.-Q. (2011). J. Phys. Chem. C.

[cit86] Li P., Zhu B., Li P., Zhang Z., Li L., Gu Y. (2019). Nanomaterials.

[cit87] Ma Y., Yan F., Liu L., Wei W., Zhao Z., Sun J. (2019). J. Photochem. Photobiol., B.

[cit88] Xiang X., Wang L., Zhang J., Cheng B., Yu J., Macyk W. (2022). Adv. Photonics Res..

[cit89] Tessier M. D., Javaux C., Maksimovic I., Loriette V., Dubertret B. (2012). ACS Nano.

[cit90] Boles M. A., Ling D., Hyeon T., Talapin D. V. (2016). Nat. Mater..

[cit91] Tyagi P., Kambhampati P. (2011). J. Chem. Phys..

[cit92] Xiang X., Zhu B., Zhang J., Jiang C., Chen T., Yu H., Yu J., Wang L. (2023). Appl. Catal., B.

[cit93] Guo X., Guo P., Wang C., Chen Y., Guo L. (2020). J. Chem. Eng..

[cit94] Kovalenko S., Schanz R., Hennig H., Ernsting N. P. (2001). J. Chem. Phys..

[cit95] Xiang X., Zhang L., Luo C., Zhang J., Cheng B., Liang G., Zhang Z., Yu J. (2024). Appl. Catal., B.

[cit96] Yan Z., Wang W., Du L., Zhu J., Phillips D. L., Xu J. (2020). Appl. Catal., B.

[cit97] Singh S., Choi S. Y., Yadav R. K., Na C. Y., Kim J., Choi M. Y., Kim T. W. (2025). Energy Fuels.

[cit98] Fathirad F., Samareh F., Bahador A. (2023). Appl. Phys. A.

[cit99] Xu S., Wang J., Jiang G., Fang Z., Lu P., Hübner R., Zhang H., Ni J., Chen F., Wang J. (2025). Adv. Funct. Mater..

[cit100] Li Q., Sritharathikhun P., Motomizu S. (2007). Anal. Sci..

[cit101] Horstkotte B., Werner E., Wiedemeier S., Elsholz O., Cerdà V., Luttmann R. (2006). Anal. Chim. Acta.

[cit102] Rakibuddin M., Kim H. (2019). Beilstein J. Nanotechnol..

[cit103] Zhao Q., Abdellah M., Cao Y., Meng J., Zou X., Ene-mark-Rasmussen K., Lin W., Li Y., Chen Y., Duan H. (2024). Adv. Funct. Mater..

[cit104] Martindale B. C., Hutton G. A., Caputo C. A., Reisner E. (2015). J. Am. Chem. Soc..

[cit105] Xu S., Wang J., Jiang G., Fang Z., Lu P., Hübner R., Zhang H., Ni J., Chen F., Wang J. (2025). Adv. Funct. Mater..

[cit106] Liu J., Liu Z., Lu P., Wang J., Lu Y., Xu S., Jiang G., Li S., Shao J., Li Z. (2026). Adv. Funct. Mater..

[cit107] Li L., Guo C., Ning J., Zhong Y., Chen D., Hu Y. (2021). Appl. Catal., B.

[cit108] Shanker G. S., Biswas A., Ogale S. (2021). JPhys Energy.

[cit109] Lin K., Qiao P., Liu Q., He Y., Kang X., Wang D., Tian C., Wu A., Fu H. (2026). Adv. Funct. Mater..

[cit110] Gawal P. M., Ishrat J., Bhattacharyya K., Golder A. K. (2025). Langmuir.

[cit111] Bhavani D. P., Kumar D. P., Rangappa A. P., Hong Y., Reddy D. A., Kim T. K. (2020). ChemCatChem.

[cit112] Naz B. B., Mubeen M., Mukhtar M., Khalid M. A., Huma Z., Waseem S., Rashid Z., Iqbal A. (2024). Proc. R. Soc. A.

