# Supplementary material for: Tuning the size of quantum dots to enhance charge transfer and photocatalytic CO2 reduction
Source: RSC Adv. 2026 Apr 13;16(21):19242–54. doi: 10.1039/d5ra08597g (PMC13075134; doi:10.1039/d5ra08597g)
Supplement: RA-016-D5RA08597G-s001 [file RA-016-D5RA08597G-s001.pdf]

Supplementary Information (SI) for RSC Advances.
This journal is © The Royal Society of Chemistry 2026

Supporting Information

Tuning Quantum Dots Size to Enhance Charge Transfer and Photocatalytic CO_2 Reduction

Muhammad Adnan Khalid[1,2], Muhammad Mubeen[1], Muhammad Nasir Hussain[1], Maria Mukhtar[1], Amna Iqbal[1], Sergey A. Kovalenko[2], Samuel Palato[2], Baljinder K. Kandola[3], Azhar Iqbal[1,3]*

[1]Department of Chemistry, Quaid-I-Azam University Islamabad-45320, Pakistan

[2]Institute of Chemistry, Humboldt University of zu Berlin,12489 Berlin, Germany

[3]Institute for Materials Research and Innovation, University of Greater Manchester, Bolton, BL3 5AB, United Kingdom

*Correspondence should be addressed to Dr. Azhar Iqbal, Email, aiqbal@qau.edu.pk

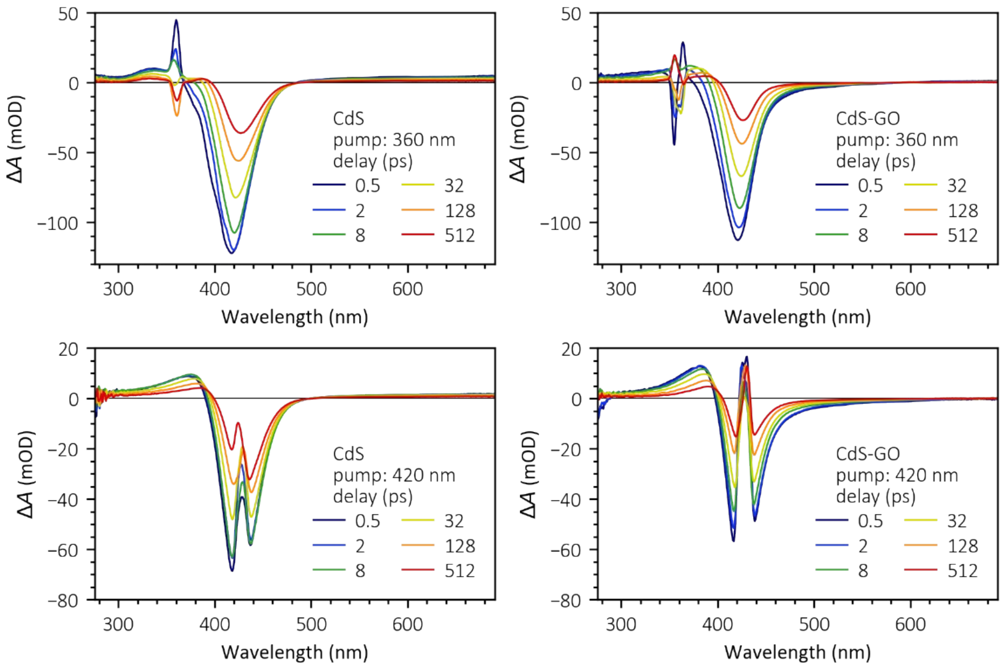

Figure S1: Comparison of TA data: excitation wavelengths (top row) 360 nm and (bottom row) 420 nm, for samples (left) CdS (right) CdS GO.
